# 3-[1-(2-Hy­droxy­anilino)ethyl­idene]-3*H*-chromen-2,4-dione

**DOI:** 10.1107/S160053681301934X

**Published:** 2013-07-24

**Authors:** Ameni Brahmia, Taicir Ben Ayed, Rached Ben Hassen

**Affiliations:** a Unité de Chimie des Matériaux et de l’Environnement, ISSBAT, Université de Tunis-ElManar, 9 Avenue Dr Zoheir SAFI, 1006 Tunis, Tunisia; b INSAT, Université de Carthage, Centre Urbain Nord, BP 676, 1080 Tunis Cedex, Tunis, Tunisia

## Abstract

The title compound is a new amino­coumarin derivative, C_17_H_13_NO_4_, and was synthesized by the condensation of 2-amino­phenol and 3-acetyl-4-hy­droxy­coumarin. An intra­molecular N—H⋯O hydrogen bond generates an *S*(6) ring motif. In the crystal, the molecules are linked into chains extending in the [010] direction by O—H⋯O hydrogen bonds. There is also a π–π stacking inter­action between the bicyclic coumarin fragment and the phenol ring [centroid–centroid distance = 3.7510 (14) Å], and these ring systems form between them a dihedral angle of 53.3 (2)°. Intermolecular hydrogen bond C—H⋯O hydrogen bonding is also observed in the interconnection of the crystal packing.

## Related literature

For related structures, see: Traven *et al.* (2000 ▶); Malecka *et al.* (2004 ▶); Mechi *et al.* (2009 ▶); Ghouili *et al.* (2011 ▶); Ketata *et al.* (2012 ▶). For the properties of coumarin derivatives, see: Bordin *et al.* (1995 ▶); Hamdi *et al.* (2010 ▶); Mahidol *et al.* (2004 ▶).
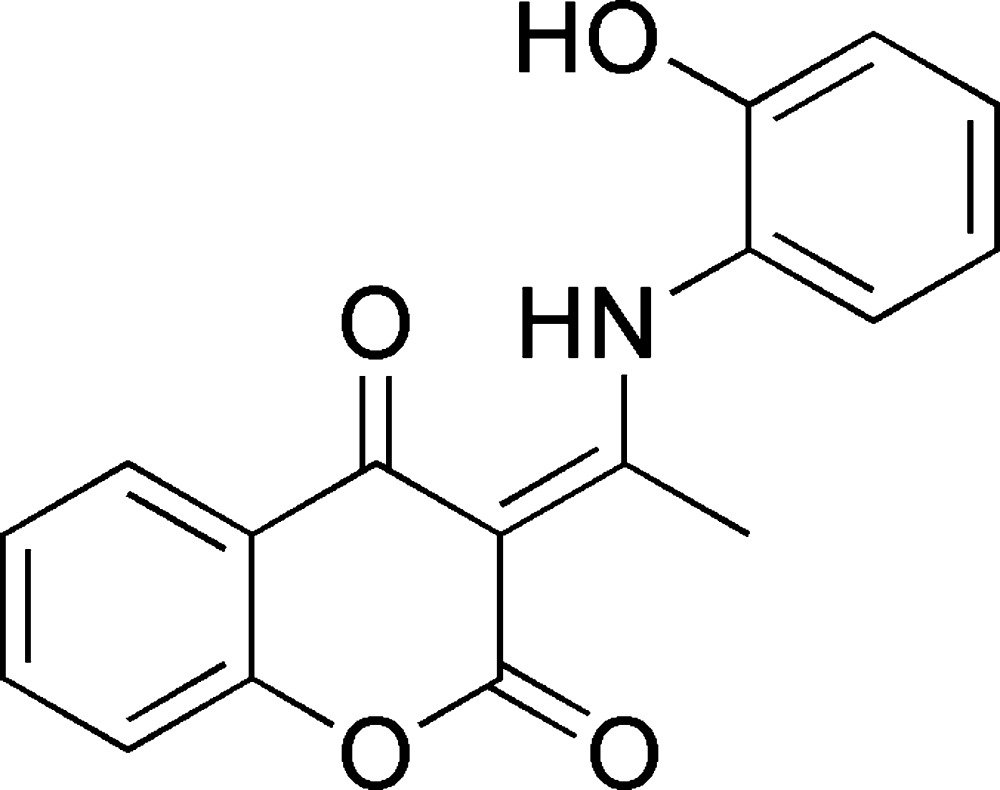


## Experimental

### 

#### Crystal data

C_17_H_13_NO_4_
*M* *_r_* = 295.29
Monoclinic, 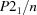

*a* = 12.5596 (4) Å
*b* = 7.5870 (3) Å
*c* = 14.3433 (6) Å
β = 94.660 (2)°
*V* = 1362.25 (9) Å^3^
*Z* = 4
Mo *K*α radiation
μ = 0.10 mm^−1^
*T* = 293 K
0.16 × 0.13 × 0.10 mm

#### Data collection

Bruker SMART CCD area-detector diffractometer
11784 measured reflections
3891 independent reflections
1721 reflections with *I* > 2σ(*I*)
*R*_int_ = 0.053

#### Refinement

*R*[*F* ^2^ > 2σ(*F* ^2^)] = 0.058
*wR*(*F* ^2^) = 0.194
*S* = 0.91
3891 reflections
251 parameters
H atoms treated by a mixture of independent and constrained refinement
Δρ_max_ = 0.23 e Å^−3^
Δρ_min_ = −0.20 e Å^−3^

### 

Data collection: *SMART* (Bruker, 2001 ▶); cell refinement: *SMART*; data reduction: *SAINT* (Bruker, 2001 ▶); program(s) used to solve structure: *SHELXS97* (Sheldrick, 2008 ▶); program(s) used to refine structure: *SHELXL97* (Sheldrick, 2008 ▶); molecular graphics: *ORTEP-3 for Windows* (Farrugia, 2012 ▶); software used to prepare material for publication: *WinGX* (Farrugia, 2012 ▶).

## Supplementary Material

Crystal structure: contains datablock(s) I, global. DOI: 10.1107/S160053681301934X/ff2111sup1.cif

Structure factors: contains datablock(s) I. DOI: 10.1107/S160053681301934X/ff2111Isup2.hkl

Supplementary material file. DOI: 10.1107/S160053681301934X/ff2111Isup3.cml

Additional supplementary materials:  crystallographic information; 3D view; checkCIF report

## Figures and Tables

**Table 1:** Hydrogen-bond geometry (Å, °)

*D*—H⋯*A*	*D*—H	H⋯*A*	*D*⋯*A*	*D*—H⋯*A*
O4—H12⋯O2^i^	0.82	1.92	2.742 (2)	175
N1—H13⋯O3	0.88 (3)	1.75 (3)	2.537 (3)	148 (3)
C8—H4⋯O2^i^	0.93	2.59	3.274 (3)	131

Symmetry code: (i) 
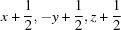
.

**Fig. 1.**
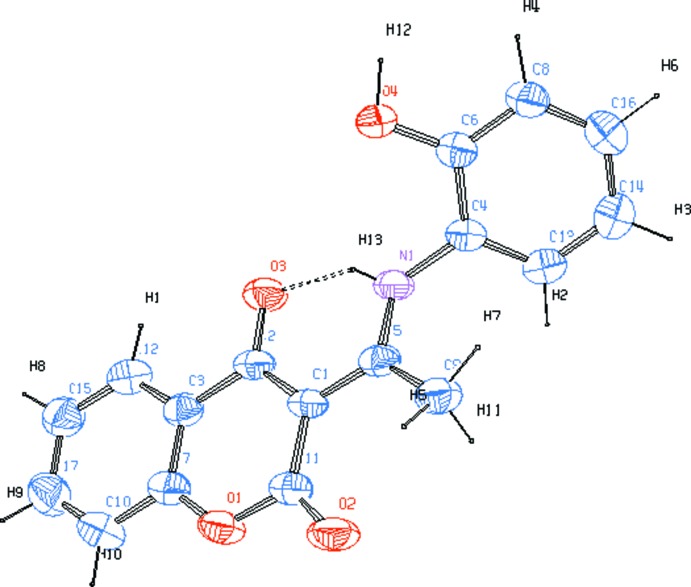
The molecular structure of the title compound showing 50% probability displacement ellipsoids and the atomic numbering. Dashed line denotes hydrogen bond.

**Fig. 2.**
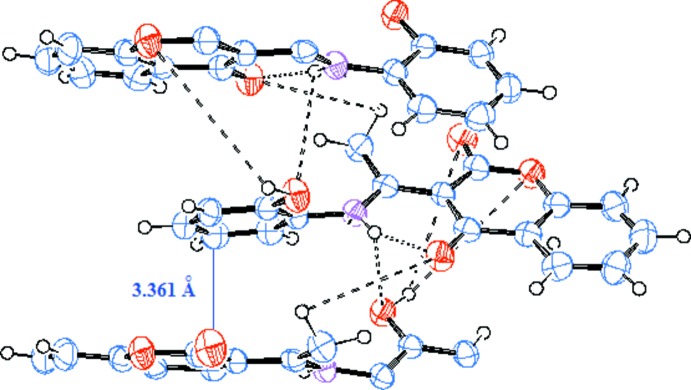
Hydrogen bonds between molecules of the title compound.

## supplementary crystallographic information

### Comment

Coumarin derivatives have a wide range of biological properties. They possess pharmacological activities, mainly anticoagulant. They also have anti-tumor, anti-oxidants and anti-inflammatory properties (Bordin *et al.*, 1995; Mahidol *et al.*, 2004). In continuation of our structural and biological studies of coumarin derivatives (Mechi *et al.*, 2009; Hamdi *et al.*, 2010; Ghouili *et al.*, 2011; Ketata *et al.*, 2012), we present the crystal structure of the title compound, a new derivative of amino coumarins.

In the crystal, the title compounds adopts a conformation where the dihedral angle between the plane of the bicyclic coumarin fragment and the phenol ring is 53.3 (2)°. The structure of our solid compound is stable thanks to the intermolecular hydrogen bonds O4—H12···O2. The crystal structure of C_17_H_13_N_1_O_4_ shows a π-π stacking interaction alternating between two inverted molecules. The stacking is observed along the *b* axis with distance 3.361 (4) Å between the layers of coumarin and phenol rings [centroid-centroid distance 3.7510 (14) Å].

The structure exhibits intramolecular hydrogen bonding N1—H13···O3, similar to that observed in amino coumarin analogue (Malecka *et al.* 2004), and we observe also a strong *p*-electron delocalization effect when comparing with O—H···O in 3-acetyl-4-hydroxycoumarin (Traven *et al.* 20000). In fact, the distances C1—C2 = 1.437 (3) Å in the title compound is longer then that one of the 3-acetyl-4-hydroxycoumarin (1.399 (1) Å) and C1—C5 = 1.428 (3) Å is shorter then 1.454 (1) Å. The elongation of N1—C5; C1—C2; C1—C11 and C11—O2 distances (see the table of bond lengths) and the shortening of O4—C6, C6—C4, C4—N1, C5—C1 and C2—O3 bond lengths, in comparison with those of the compound C_13_H_13_N_1_O_4_ (Malecka *et al.* 2004) could be related to the electron-donor effect of the phenolic group in the title compound.

The linkage between the coumarin system and aminophenol ring exhibits bond lengths O3—C2 = 1.251 (3) Å, C2—C1 = 1.437 (3) Å, C1—C5 = 1.428 (3) Å, C5—N1 = 1.316 (3) Å and N1—C4 = 1.422 (3) Å, suggesting that all non-hydrogen atoms between the electron-donors and acceptors are highly conjugated, leading to a π-bridge for the charge transfer from aminophenol ring to coumarin system.

### Experimental

The amino coumarin was synthesized by the condensation of an equimolar amount of 2-aminophenol and 3-acetyl-4-hydroxycoumarin in absolute ethanol. After four hours of reflux, the reaction mixture was left crystallizing at room temperature. The compound obtained is presented as transparent crystals of light yellow color with shape and size suitable for the structural study of X-ray single-crystal. Yield:(90%). mp= 446 K. IR: ν 3155 (NH), 2973 (OH), 1666 (>C=O), 1609 (C=C), 1102 (C—O); 1HNMR: δp.p.m.: 2.54 (s, 3H, Hmethyl), 6.51–7.14 (m, 4H, Ar—H), 7.23–8.12 (m, 4H, Ph—H), 10.45 (s, 1H, OH), 15.20 (s, 1H, NH); 13CNMR: (p.p.m.): 20.30 (C methyl), 97.11 (C3), 116.27–134.34 (C arom), 151.40 (C—OH), 161.66 (C=O lactone), 175.90 (C—N), 180.19 (C=O ketone),

### Refinement

The hydrogen atoms are fixed geometrically and refined as riding with the exception of the H13, which was located from electron density difference map and is refined isotropically. *U*_iso_(H) values of the H atoms were set at 1.2*U*_eq_ or 1.5*U*_eq_ of the parent atom.

### Crystal data

**Table:**

C_17_H_13_NO_4_	*F*(000) = 616
*M**_r_* = 295.29	*D*_x_ = 1.440 Mg m^−^^3^
Monoclinic, *P*2_1_/*n*	Melting point: 446 K
Hall symbol: -P2yn	Mo *K*α radiation, λ = 0.71073 Å
*a* = 12.5596 (4) Å	Cell parameters from 1721 reflections
*b* = 7.5870 (3) Å	µ = 0.10 mm^−^^1^
*c* = 14.3433 (6) Å	*T* = 293 K
β = 94.660 (2)°	Needle, yellow
*V* = 1362.25 (9) Å^3^	0.16 × 0.13 × 0.10 mm
*Z* = 4	

### Data collection

**Table:**

Bruker SMART CCD area-detector diffractometer	1721 reflections with *I* > 2σ(*I*)
Radiation source: fine-focus sealed tube	*R*_int_ = 0.053
Graphite monochromator	θ_max_ = 29.8°, θ_min_ = 2.1°
φ and ω scans	*h* = −14→17
11784 measured reflections	*k* = −9→10
3891 independent reflections	*l* = −19→20

### Refinement

**Table:**

Refinement on *F*^2^	Primary atom site location: structure-invariant direct methods
Least-squares matrix: full	Secondary atom site location: difference Fourier map
*R*[*F*^2^ > 2σ(*F*^2^)] = 0.058	Hydrogen site location: inferred from neighbouring sites
*wR*(*F*^2^) = 0.194	H atoms treated by a mixture of independent and constrained refinement
*S* = 0.91	*w* = 1/[σ^2^(*F*_o_^2^) + (0.0922*P*)^2^ + 0.2201*P*] where *P* = (*F*_o_^2^ + 2*F*_c_^2^)/3
3891 reflections	(Δ/σ)_max_ < 0.001
251 parameters	Δρ_max_ = 0.23 e Å^−^^3^
0 restraints	Δρ_min_ = −0.20 e Å^−^^3^

### Special details

**Table:**

Geometry. All e.s.d.'s (except the e.s.d. in the dihedral angle between two l.s. planes) are estimated using the full covariance matrix. The cell e.s.d.'s are taken into account individually in the estimation of e.s.d.'s in distances, angles and torsion angles; correlations between e.s.d.'s in cell parameters are only used when they are defined by crystal symmetry. An approximate (isotropic) treatment of cell e.s.d.'s is used for estimating e.s.d.'s involving l.s. planes.
Refinement. Refinement of *F*^2^ against ALL reflections. The weighted *R*-factor *wR* and goodness of fit *S* are based on *F*^2^, conventional *R*-factors *R* are based on *F*, with *F* set to zero for negative *F*^2^. The threshold expression of *F*^2^ > σ(*F*^2^) is used only for calculating *R*-factors(gt) *etc*. and is not relevant to the choice of reflections for refinement. *R*-factors based on *F*^2^ are statistically about twice as large as those based on *F*, and *R*- factors based on ALL data will be even larger.

### Fractional atomic coordinates and isotropic or equivalent isotropic displacement parameters (Å^2^)

**Table:**

	*x*	*y*	*z*	*U*_iso_*/*U*_eq_	
O1	0.12600 (13)	0.1466 (2)	0.90032 (12)	0.0480 (5)	
N1	0.22554 (16)	0.0473 (3)	1.22516 (14)	0.0375 (5)	
C1	0.17836 (17)	0.0955 (3)	1.06478 (16)	0.0350 (5)	
O3	0.34691 (12)	−0.0400 (2)	1.09988 (12)	0.0484 (5)	
C2	0.27686 (18)	0.0180 (3)	1.04068 (17)	0.0378 (6)	
C3	0.29714 (18)	0.0128 (3)	0.94130 (17)	0.0389 (6)	
C4	0.21353 (18)	0.0298 (3)	1.32227 (17)	0.0368 (6)	
O4	0.38154 (13)	0.1659 (2)	1.35092 (12)	0.0473 (5)	
H12	0.4244	0.1965	1.3941	0.071*	
C5	0.15554 (17)	0.1107 (3)	1.16048 (17)	0.0363 (6)	
O2	0.01364 (14)	0.2201 (3)	1.00033 (13)	0.0556 (5)	
C6	0.29532 (19)	0.0900 (3)	1.38614 (18)	0.0376 (6)	
C7	0.22130 (19)	0.0795 (3)	0.87552 (18)	0.0417 (6)	
C8	0.2850 (2)	0.0704 (3)	1.48055 (18)	0.0439 (6)	
H4	0.3384	0.1118	1.5238	0.053*	
C9	0.05868 (19)	0.2032 (4)	1.19047 (19)	0.0475 (7)	
H5	0.0586	0.1977	1.2573	0.071*	
H11	−0.0045	0.1470	1.1623	0.071*	
H7	0.0598	0.3242	1.1710	0.071*	
C10	0.2392 (2)	0.0838 (4)	0.78132 (19)	0.0546 (7)	
H10	0.1871	0.1274	0.7375	0.065*	
C11	0.10187 (19)	0.1569 (3)	0.99164 (18)	0.0416 (6)	
C12	0.3922 (2)	−0.0510 (3)	0.9114 (2)	0.0481 (7)	
H1	0.4431	−0.0990	0.9548	0.058*	
C13	0.12623 (19)	−0.0559 (3)	1.35386 (19)	0.0451 (7)	
H2	0.0735	−0.1007	1.3111	0.054*	
C14	0.1168 (2)	−0.0752 (4)	1.4483 (2)	0.0503 (7)	
H3	0.0575	−0.1314	1.4695	0.060*	
C15	0.4121 (2)	−0.0446 (4)	0.8187 (2)	0.0545 (7)	
H8	0.4768	−0.0848	0.7996	0.065*	
C16	0.1960 (2)	−0.0104 (4)	1.51091 (19)	0.0497 (7)	
H6	0.1893	−0.0214	1.5747	0.060*	
C17	0.3343 (2)	0.0230 (4)	0.7537 (2)	0.0583 (8)	
H9	0.3471	0.0268	0.6908	0.070*	
H13	0.283 (2)	0.007 (4)	1.201 (2)	0.061 (9)*	

### Atomic displacement parameters (Å^2^)

**Table:**

	*U*^11^	*U*^22^	*U*^33^	*U*^12^	*U*^13^	*U*^23^
O1	0.0451 (10)	0.0600 (12)	0.0366 (10)	0.0120 (9)	−0.0099 (8)	0.0012 (9)
N1	0.0327 (11)	0.0452 (12)	0.0330 (11)	0.0007 (9)	−0.0072 (9)	−0.0010 (9)
C1	0.0327 (11)	0.0370 (13)	0.0335 (13)	0.0004 (10)	−0.0089 (10)	0.0011 (10)
O3	0.0378 (9)	0.0621 (12)	0.0433 (10)	0.0091 (8)	−0.0085 (8)	0.0041 (9)
C2	0.0347 (12)	0.0368 (13)	0.0398 (13)	−0.0011 (10)	−0.0104 (10)	0.0017 (11)
C3	0.0387 (13)	0.0355 (13)	0.0413 (14)	−0.0040 (11)	−0.0045 (11)	−0.0031 (11)
C4	0.0355 (12)	0.0391 (13)	0.0342 (13)	0.0009 (10)	−0.0057 (10)	−0.0007 (11)
O4	0.0390 (9)	0.0621 (12)	0.0390 (10)	−0.0135 (8)	−0.0080 (8)	0.0009 (9)
C5	0.0339 (12)	0.0330 (13)	0.0403 (14)	−0.0055 (10)	−0.0082 (10)	−0.0012 (10)
O2	0.0406 (10)	0.0748 (14)	0.0484 (11)	0.0151 (9)	−0.0141 (8)	0.0016 (10)
C6	0.0403 (13)	0.0340 (13)	0.0375 (13)	−0.0002 (10)	−0.0032 (10)	−0.0001 (11)
C7	0.0436 (14)	0.0406 (14)	0.0393 (14)	−0.0002 (11)	−0.0069 (11)	−0.0026 (11)
C8	0.0474 (14)	0.0477 (16)	0.0351 (14)	−0.0028 (12)	−0.0063 (11)	−0.0026 (12)
C9	0.0409 (13)	0.0541 (17)	0.0462 (16)	0.0054 (12)	−0.0047 (12)	−0.0015 (13)
C10	0.0630 (18)	0.0593 (18)	0.0395 (16)	0.0063 (15)	−0.0078 (13)	0.0033 (13)
C11	0.0399 (13)	0.0428 (15)	0.0400 (15)	−0.0012 (12)	−0.0094 (11)	0.0003 (12)
C12	0.0413 (13)	0.0493 (16)	0.0519 (17)	0.0012 (12)	−0.0073 (12)	−0.0047 (13)
C13	0.0377 (13)	0.0486 (16)	0.0475 (16)	−0.0029 (11)	−0.0056 (12)	−0.0019 (12)
C14	0.0471 (15)	0.0493 (16)	0.0554 (18)	−0.0022 (12)	0.0104 (13)	0.0016 (14)
C15	0.0489 (15)	0.0655 (19)	0.0492 (17)	−0.0035 (14)	0.0040 (13)	−0.0079 (15)
C16	0.0601 (17)	0.0510 (16)	0.0384 (14)	0.0040 (14)	0.0057 (13)	0.0006 (13)
C17	0.0656 (18)	0.067 (2)	0.0427 (16)	−0.0056 (16)	0.0035 (14)	−0.0031 (15)

### Geometric parameters (Å, º)

**Table:**

O1—C7	1.374 (3)	C7—C10	1.388 (4)
O1—C11	1.370 (3)	C8—C16	1.376 (4)
N1—C5	1.316 (3)	C8—H4	0.9300
N1—C4	1.419 (3)	C9—H5	0.9600
N1—H13	0.88 (3)	C9—H11	0.9600
C1—C5	1.429 (3)	C9—H7	0.9600
C1—C2	1.437 (3)	C10—C17	1.369 (4)
C1—C11	1.441 (3)	C10—H10	0.9300
O3—C2	1.252 (3)	C12—C15	1.373 (4)
C2—C3	1.469 (3)	C12—H1	0.9300
C3—C7	1.381 (3)	C13—C14	1.376 (4)
C3—C12	1.388 (3)	C13—H2	0.9300
C4—C13	1.382 (3)	C14—C16	1.376 (4)
C4—C6	1.396 (3)	C14—H3	0.9300
O4—C6	1.359 (3)	C15—C17	1.393 (4)
O4—H12	0.8200	C15—H8	0.9300
C5—C9	1.498 (3)	C16—H6	0.9300
O2—C11	1.223 (3)	C17—H9	0.9300
C6—C8	1.379 (3)		
			
C7—O1—C11	122.26 (19)	C5—C9—H11	109.5
C5—N1—C4	127.4 (2)	H5—C9—H11	109.5
C5—N1—H13	112 (2)	C5—C9—H7	109.5
C4—N1—H13	121 (2)	H5—C9—H7	109.5
C5—C1—C2	120.5 (2)	H11—C9—H7	109.5
C5—C1—C11	119.9 (2)	C17—C10—C7	119.2 (3)
C2—C1—C11	119.6 (2)	C17—C10—H10	120.4
O3—C2—C1	123.5 (2)	C7—C10—H10	120.4
O3—C2—C3	118.8 (2)	O2—C11—O1	113.1 (2)
C1—C2—C3	117.7 (2)	O2—C11—C1	127.5 (2)
C7—C3—C12	118.6 (2)	O1—C11—C1	119.4 (2)
C7—C3—C2	119.3 (2)	C15—C12—C3	121.0 (3)
C12—C3—C2	122.0 (2)	C15—C12—H1	119.5
C13—C4—C6	120.0 (2)	C3—C12—H1	119.5
C13—C4—N1	121.1 (2)	C4—C13—C14	120.4 (2)
C6—C4—N1	118.8 (2)	C4—C13—H2	119.8
C6—O4—H12	109.5	C14—C13—H2	119.8
N1—C5—C1	118.2 (2)	C13—C14—C16	119.3 (2)
N1—C5—C9	118.7 (2)	C13—C14—H3	120.4
C1—C5—C9	123.0 (2)	C16—C14—H3	120.4
O4—C6—C8	123.5 (2)	C12—C15—C17	119.3 (3)
O4—C6—C4	117.4 (2)	C12—C15—H8	120.4
C8—C6—C4	119.1 (2)	C17—C15—H8	120.4
O1—C7—C3	121.7 (2)	C8—C16—C14	121.0 (3)
O1—C7—C10	117.2 (2)	C8—C16—H6	119.5
C3—C7—C10	121.1 (2)	C14—C16—H6	119.5
C6—C8—C16	120.1 (2)	C10—C17—C15	120.7 (3)
C6—C8—H4	119.9	C10—C17—H9	119.6
C16—C8—H4	119.9	C15—C17—H9	119.6
C5—C9—H5	109.5		

### Hydrogen-bond geometry (Å, º)

**Table:**

*D*—H···*A*	*D*—H	H···*A*	*D*···*A*	*D*—H···*A*
O4—H12···O2^i^	0.82	1.92	2.742 (2)	175
N1—H13···O3	0.88 (3)	1.75 (3)	2.537 (3)	148 (3)
C8—H4···O2^i^	0.93	2.59	3.274 (3)	131

Symmetry code: (i) *x*+1/2, −*y*+1/2, *z*+1/2.
